# Changes in expression profiles of internal jugular vein wall and plasma protein levels in multiple sclerosis

**DOI:** 10.1186/s10020-018-0043-4

**Published:** 2018-08-09

**Authors:** Giovanna Marchetti, Nicole Ziliotto, Silvia Meneghetti, Marcello Baroni, Barbara Lunghi, Erica Menegatti, Massimo Pedriali, Fabrizio Salvi, Ilaria Bartolomei, Sofia Straudi, Fabio Manfredini, Rebecca Voltan, Nino Basaglia, Francesco Mascoli, Paolo Zamboni, Francesco Bernardi

**Affiliations:** 1 Department of Biomedical and Specialty Surgical Sciences, University of Ferrara, via Fossato di Mortara n 74, 44121 Ferrara, Italy; 2 Department of Life Science and Biotechnology, University of Ferrara, Ferrara, Italy; 3 Department of Morphology, Surgery and Experimental Medicine, University of Ferrara, Ferrara, Italy; 4 Department of Experimental and Diagnostic Medicine, Sant’Anna University- Hospital, Ferrara, Italy; 5 Center for Immunological and Rare Neurological Diseases, Bellaria Hospital, IRCCS of Neurological Sciences, Bologna, Italy; 6 Department of Neurosciences and Rehabilitation, Sant’Anna University- Hospital, Ferrara, Italy; 7 Unit of Vascular and Endovascular Surgery, S. Anna University-Hospital, Ferrara, Italy

**Keywords:** Gene expression, Jugular vein wall, Multiple sclerosis, Chronic cerebrospinal venous insufficiency, Venous abnormalities, Jugular plasma protein levels, Multiplex protein assay, Chemokines, Adhesion molecules

## Abstract

**Background:**

Multiple sclerosis (MS) is an inflammatory, demyelinating and degenerative disorder of the central nervous system (CNS). Several observations support interactions between vascular and neurodegenerative mechanisms in multiple sclerosis (MS). To investigate the contribution of the extracranial venous compartment, we analysed expression profiles of internal jugular vein (IJV), which drains blood from CNS, and related plasma protein levels.

**Methods:**

We studied a group of MS patients (*n* = 19), screened by echo-color Doppler and magnetic resonance venography, who underwent surgical reconstruction of IJV for chronic cerebrospinal venous insufficiency (CCSVI). Microarray-based transcriptome analysis was conducted on specimens of IJV wall from MS patients and from subjects undergoing carotid endarterectomy, as controls. Protein levels were determined by multiplex assay in: i) jugular and peripheral plasma from 17 MS/CCSVI patients; ii) peripheral plasma from 60 progressive MS patients, after repeated sampling and iii) healthy individuals.

**Results:**

Of the differentially expressed genes (≥ 2 fold-change, multiple testing correction, *P* < 0.05), the immune-related *CD86* (8.5 fold-change, *P* = 0.002) emerged among the up regulated genes (*N* = 409). Several genes encoding HOX transcription factors and histones potentially regulated by blood flow, were overexpressed. Smooth muscle contraction and cell adhesion processes emerged among down regulated genes (*N* = 515), including the neuronal cell adhesion *L1CAM* as top scorer (5 fold-change, *P* = 5 × 10^− 4^).

Repeated measurements in jugular/peripheral plasma and overtime in peripheral plasma showed conserved individual plasma patterns for immune-inflammatory (CCL13, CCL18) and adhesion (NCAM1, VAP1, SELL) proteins, despite significant variations overtime (SELL *P* < 0.0001). Both age and MS disease phenotypes were determinants of VAP1 plasma levels.

Data supported cerebral related-mechanisms regulating ANGPT1 levels, which were remarkably lower in jugular plasma and correlated in repeated assays but not between jugular/peripheral compartments.

**Conclusions:**

This study provides for the first time expression patterns of the IJV wall, suggesting signatures of altered vascular mRNA profiles in MS disease also independently from CCSVI. The combined transcriptome-protein analysis provides intriguing links between IJV wall transcript alteration and plasma protein expression, thus highlighting proteins of interest for MS pathophysiology.

**Electronic supplementary material:**

The online version of this article (10.1186/s10020-018-0043-4) contains supplementary material, which is available to authorized users.

## Background

Multiple sclerosis (MS) is an inflammatory demyelinating and degenerative disorder of the central nervous system (CNS) (Noseworthy et al. 2000) for which several genetic, epigenetic and environmental components have been proposed to participate through complex interactions (Amato et al. 2018; Olsson et al. 2017).

Several observations suggest that vascular components are involved in the multifactorial pathogenetic interplay and/ or in disease progression, severity and comorbidities development (Karmon et al. 2012; Spencer et al. 2018; Kappus et al. 2016).

The vascular cerebral system, and particularly the venous compartment, early received attention because of venous thrombosis in the brain of MS patients, and plaques of demyelination development around venules and perivascular infiltrations of inflammatory cells just next small and medium size venous of CNS (Adams 1988).

The condition named chronic cerebrospinal venous insufficiency (CCSVI) provided the possible association of MS with extra-cranial venous abnormalities which impaired venous outflow (Zamboni et al. 2009; Zivadinov et al. 2012).

Although highly debated whether associated with MS, and not leading to a viable treatment option in patients (Zamboni et al. 2018), this condition favors better understanding of the function and role of the extracranial venous system in MS (Zivadinov and Weinstock-Guttman 2018). On the other hand, a perspective of reduced blood supply to the brain (D'haeseleer et al. 2015), further argue for the relevance of the vascular component in the disease.

Findings on these conditions associated to MS foster more investigations of both intracranial and extracranial vascular compartments changes in MS (Zivadinov and Weinstock-Guttman 2018; Belov et al. 2018).

Vascular features associated to MS have been deeply investigated (D'haeseleer et al. 2011; Dolic et al. 2012), with the central vein sign recently proposed as a MRI biomarker of MS (Sati et al. 2016). Studies focusing on circulating and endothelial components, which participate in the complex network of immune-vascular interactions have been reported (Alexander et al. 2013). De-regulated patterns of gene expression have been detected in peripheral whole blood or peripheral blood mononuclear cells (PBMC) of MS patients (Ramanathan et al. 2001; Ratzer et al. 2013; Nickles et al. 2013; Paraboschi et al. 2015; Comabella et al. 2016; Lindsey et al. 2011).

To shed light on vascular gene expression changes in MS with associated CCSVI, we focused on internal jugular vein (IJV), which drains blood from the brain. In particular, we explored gene expression changes by using two informative approaches and their combination, transcriptomic analysis on IJV specimens and specific protein assays on plasma from both jugular and peripheral veins.

## Methods

### Study populations

The first (1st) study population was represented by a group of 19 Italian subjects with MS and positive screening for CCSVI.

Diagnosis of MS was in accordance to the McDonald criteria (Polman et al. 2005). Patients’ screening through flow quantification by means of a combination of validated echo-color Doppler (ECD) model with magnetic resonance venography morphological and flow evaluation protocol, and cerebral perfusion evaluation by SPECT-CT, have been previously detailed (Dolic et al. 2012; Zamboni et al. 2013; Zamboni et al. 2016). The patients presented truncular venous malformation in at least one IJV, in form of segmental hypoplasia, defective valves with incomplete or absent opening of their leaflets, other intraluminal obstacles and muscular compression. The 19 patients belonged to a cohort of patients who were eligible for surgical reconstruction of internal jugular vein by angioplasty and entered the study approved by the Ethical Committee of the S. Anna University-Hospital of Ferrara. The details about enrolment and inclusion/exclusion criteria have been previously described (Zamboni et al. 2016). 1st MS population demographics are reported in Table 1.

**Table 1:** First study population demographics

	MS-CCSVI Patients *n* = 19	Healthy subjects *n* = 34
Age, mean ± SD	46.5 ± 8.6	41.3 ± 9
Gender, M/F	10/9	13/21
MS clinical class		
RR	11	–
SP	7	
PP	1	
Disease duration RR, mean ± SD	10 ± 4	–
Disease duration SP – PP, mean ± SD	13 ± 4	–
EDSS, mean ± SD	4 ± 2	–
MRI T1 gadolinium enhancing lesions, n	5/19	–
M-mode IJV defective valves, n	29/38	–

*RR* relapsing remitting, *SP* secondary progressive, *PP* primary progressive, *EDSS* expanded disability status scale, *M-mode* echo Doppler. Age and disease duration are reported in years

The second (2nd) study population included 60 Italian MS patients, who participated in the RAGTIME study (ClinicalTrials.gov ID:NCT02421731) (Straudi et al. 2017). This clinical trial compares robot-assisted gait training versus conventional therapy on mobility in severely disabled progressive MS patients. The demographics and clinical characteristics of the 2nd MS population are reported in Additional file 1: Table S1.

Thirty-four Italian healthy subjects (mean age 41.3 ± 9.0; 21 women and 13 men), who have never diagnosed with MS, neurological disorder or other chronic inflammatory diseases, were recruited for protein level analysis in plasma. Eight healthy subjects were recruited and added to the healthy cohort (total subjects = 42; mean age 41.29 ± 11.4; 26 women and 16 men) as control group for the 2nd MS population.

### Jugular wall specimens

IJV specimens were obtained at surgery from patients. In MS patients, the surgical procedure included an unilateral or bilateral supra-clavicular transverse incision of about 5 cm. The IJV was isolated at the junction with the subclavian vein. The latter was tangentially clamped following systemic injection of heparin. An endo-phlebectomy was subsequently performed with complete removal of the jugular valve/septum and of a tiny specimen of jugular wall, followed by a patch angioplasty using the autologous great saphenous vein. Omohyoid muscle section was performed, if the pre-operative finding of extrinsic compression was confirmed in the surgical theatre.

Control IJV specimens were obtained from patients without MS or other neurological diseases, undergoing carotid endarterectomy (CEA) for high-grade carotid stenosis. In these five patients ECD analysis of carotid, vertebral and subclavian arteries, and jugular veins, documented the presence of atherosclerotic plaque, mostly localized at carotid bifurcation, and did not detect jugular vein alterations.

During the CEA procedure, the access to common carotid artery needs to separate the small facial vein, crossing the carotid artery just at the level of bifurcation, from the jugular vein. A very small full thickness specimen of jugular wall was taken during this maneuver.

Written informed consent was obtained from all subjects.

Specimens retrieved at surgery were immediately placed into RNAlater (Ambion Inc., Austin, TX) and then stored at − 80 °C.

### Microarray-based transcriptome analysis of jugular vein walls

From homogenized wall specimens (TRIZOL Reagent, Invitrogen Carlsab, CA), total RNA was extracted using the miRNeasy Mini Kit (Quiagen, Hilden, Germany) and its quality was assessed with Agilent 2100 Bioanalyzer (Agilent Technologies, Palo Alto, CA). Labelled cRNA was synthesized from 100 ng of total RNA using the Low RNA Input Linear Amplification Kit (Agilent Technologies) in the presence of cyanine 3-CTP (Perkin-Elmer Life Sciences, Boston, MA). Hybridization on Agilent whole human genome oligo microarray (Cat.No. G4851A, Agilent Technologies), which represents 60,000 unique human transcripts, was performed in accordance to manufacturer’s indications.

Microarray raw-data were obtained with Feature Extraction software v.10.7 (Agilent Technologies) and analyzed by using the GeneSpring GX v.14 software (Agilent Technologies) as previously described (Coen et al. 2013a; Marchetti et al. 2015).

### cDNA preparation and quantitative real-time polymerase chain reaction (qRT-PCR)

cDNA was obtained from 0.150 μg of total RNA by reverse transcription using M-MLV Reverse Transcriptase (Invitrogen Carlsab, CA) and a mixture of oligo(dT) and random primers.

Aliquots of diluted cDNA were amplified using SsoFast EvaGreen Supermix (BioRad, Hercules, CA).

As general approach for qRT-PCR the specific primers were chosen to amplify the regions recognized by oligonucleotide probes in the microarray analysis. Forward and reverse primers are reported in the Additional file 2: Table S2. PCR protocol was: 95 °C for 30 s, then 40 cycles of 10 s at 95 °C and 15 s at 58 °C. Each reaction was performed in triplicate. All qRT-PCRs were performed on an CFX96 Real-Time PCR Detection System instrument (BioRad, Hercules, CA) according to the manufacturer’s instructions. The relative levels of mRNAs were calculated by 2^-ΔΔCt^ method using *ACTB* and *B2M* as endogenous controls. Values were expressed as mean fold change ± standard error of the mean.

### Plasma samples

For the 1st MS population (*N* = 17 patients) blood samples were drawn during the surgical procedure, before systemic injection of heparin, from both IJV (right or left) and a peripheral vein. At time of blood sampling patients were free of therapy for at least one month. All blood samples were drawn at fasting in citrate tubes.

MS patients from the 2nd population, enrolled in the RAGTIME study, provided blood sampling at four time points: T0) baseline point, prior to the first rehabilitative session; T1) intermediate point, after six training sessions; T2) end of treatment, 12 completed rehabilitative sessions, 1 month after T0; T3) follow-up, after 3 months from the end of training program (30)(Ziliotto et al. 2018).

Peripheral venous blood samples were also collected from the healthy volunteers (*n* = 34, or plus eight subjects *n* = 42, as control group for the 2nd MS population).

All plasma samples were separated by two centrifugations (15 min at 2500 g and 5 min at 11000 g at room temperature), aliquoted and frozen at − 80 °C until use.

Protein antigen levels were quantified by a custom-designed Luminex Screening Assays magnetic bead kits (Luminex R&D Systems Inc., Minneapolis, MN, USA) according to the manufacturer’s instructions. Data were acquired using the Luminex® 100 system and analyzed using Bioplex Manager Software version 6.0 (both from Biorad Laboratories, Hercules, CA). Soluble CD86 was measured by an enzyme-linked immunosorbent (ELISA)-based assay according to the manufacturer’s protocol (Abcam, Cambridge, UK). The inter assay variability assessed by using coefficients of variation (CV%) were as follows: 1.1 (NCAM1), 1.2 (ANGPT1), 2.1 (CCL13), 2.3 (VAP1), 2.6 (CCL18), 3 (SELL), and 3.2 (CD86).

### Statistical analysis

In tissue microarray a filter on low gene expression was used to keep only the probes expressed in at least one sample (flagged as Marginal or Present). Then, samples were grouped in accordance to their disease status (MS and Controls) and compared. To evaluate similarities or differences among each group (MS and Controls) principal component analysis was performed on the normalized data using the GeneSpring GX v.14 software (Agilent Technologies). Differentially expressed genes were selected as having a 2-fold expression difference between their geometrical mean in the two groups and a statistically significant *p*-value (< 0.05) by a moderate t-test, followed by the application of Benjamini- Hoechberg multiple testing correction.

Differentially expressed genes were employed for Cluster Analysis of samples, using the Manhattan correlation as a measure of similarity. Functional categorization was assigned using Gene Ontology (GO) by free access DAVID Bioinformatics database 6.7. Gene expression levels between MS and control jugular walls in qRT-PCR analysis were compared by means of unpaired t-test.

Protein plasma levels were expressed as mean ± SD. Differences between plasma sample groups were assessed by paired or unpaired Student’s t test and by ANCOVA test using age as covariate. A *p*-value ≤ 0.05 was considered statistically significant. Pearson’s test was used to assess correlation between jugular and peripheral plasma levels in MS patients and to assess correlation over time for ANGPT1, CCL13, CCL18, NCAM1, SELL and VAP1 plasma levels. ANOVA for repeated measures was used to test differences across the four time points and, in case of a significant p-value, pairwise comparisons were Bonferroni corrected (q-values). All statistical analyses were performed using IBM® SPSS® Statistics version 24 software (IBM Corp. Armonk, NY, USA).

## Results

### Analysis of gene expression profiles in jugular vein specimens

To explore the expression pattern of IJV wall of patients with MS as compared to unaffected jugular walls, total RNAs extracted from MS specimens (*n* = 4) and from control specimens (*n* = 5) were subjected to microarray analysis. Using the criteria of at least a 2-fold difference in the expression level (corrected *P* value < 0.05) between the two groups of RNA samples (see Methods), a total of 924 transcripts were found to be differentially expressed (Additional file 3: Table S3).

**Fig. 1:**
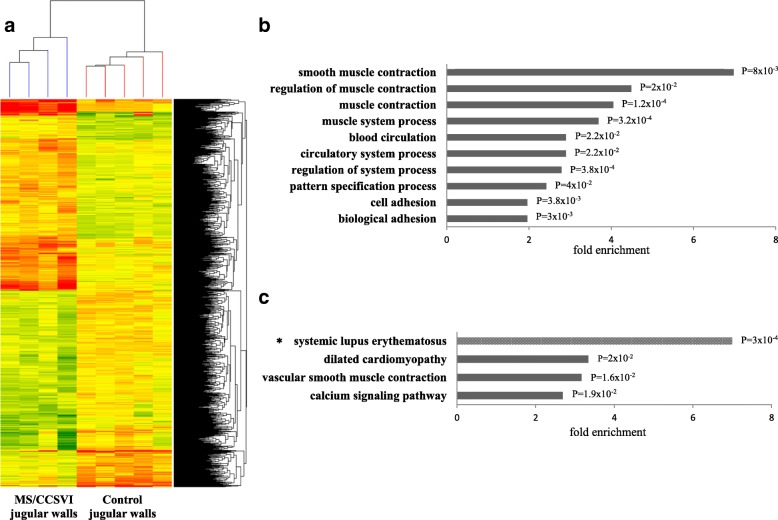
Transcriptomic analysis in internal jugular vein walls. **a** Heat map representation of the 924 differentially expressed genes (1408 probes). Each column represents one RNA sample (MS/CCSVI and control jugular walls) and each row represents one gene (probe). Colors represent the expression level fold change: higher-red, lower- green and no difference-yellow. **b** Enriched biological processes and (**c**) pathways associated to the 924 genes differentially expressed between MS and control jugular walls. Significantly overrepresented terms (Benjamini test *P* < 0.05) were selected from DAVID bioinformatics 6.7 by the Functional Annotation Chart resource. *Pathway significantly overrepresented only among up-regulated genes

Clustering analysis (Fig. 1a) indicated that 409 transcripts were up- and 515 down- regulated in MS J wall. Testing for RNA function in NCBI database showed that up regulation was observed for 300 coding and 109 non-coding or uncharacterized RNAs, whereas down regulation was detected for 429 coding and 86 non-coding RNAs.

To assign a functional annotation to the 924 transcripts/genes, GO analysis by the Functional Annotation Chart Instrument of DAVID Bioinformatics 6.7 was conducted. The most enriched biological processes (*P* < 0.05, Benjamini test) are reported in Fig. 1b. In this selection the highest significance was related to the terms “muscle”/“smooth muscle contraction” (*P* = 1.2 × 10^− 4^ and *P* = 0.008 respectively) and “biological adhesion” (*P* = 0.003), with the term “smooth muscle contraction” showing the highest fold enrichment (7.35).

When sub-analysing up- and down- regulated transcripts (*P* < 0.05, Benjamini test), the terms “pattern specification process” and “nucleosome organization” were overrepresented among up-regulated genes. The biological process “pattern specification” included several homeobox (HOX) genes (*HOXA5, HOXA6, HOXA7, HOXB5, HOXB6, HOXC4* and *HOXC5*), which encode for transcription factors. The GO term “nucleosome organization” comprised several histone subunit genes, and in particular, three H3 variant genes (*HIST1H3D, HIST1H3F* and *HIST1H3H*) were included in the list of the most significantly (*P* = 0.002) up-regulated coding RNAs. Among the down regulated genes, the terms related to smooth muscle contraction showed the highest fold enrichment (12.0). Concerning the enriched adhesion terms, 51 genes were found downregulated, of which *L1CAM,* encoding for the neural cell adhesion molecule L1, was included among the top 10 most significantly downregulated genes (*P* = 5 × 10^− 4^).

To gain further insight into functional associations, analysis of KEGG and BIOCARTA pathways by DAVID Bioinformatics resource for the 924 transcripts/genes was performed. The selection for enrichment (*P* < 0.05, Benjamini test) provided the terms “vascular smooth muscle contraction”, “dilated cardiomyopathy” and “calcium signaling pathway” (Fig. 1c). Among the up- regulated genes, the only enriched term (fold enrichment 7.0, *P* = 3 × 10^− 4^) was the “systemic lupus erythematosus” pathway (Fig. 1c).

### Expression analysis by quantitative real time PCR (qRT-PCR)

Aimed at supporting microarray profiling results by a different assay, qRT-PCR analyses were performed on additional jugular wall samples of MS patients (*N* = 7) and of controls (*N* = 4). Five genes were selected (*ANGPT1*, *AOC3*, *CD86*, *L1CAM* and *SELL*), three of which included in the biological process “adhesion”. Three genes were also included in the list of the top ten most significantly up regulated (*CD86*) or down regulated (*L1CAM, ANGPT1*) genes (Additional file 3: Table S3).

Significant differences (Additional file 4: Table S4) were observed for *L1CAM* (*ACTB*, *P* = 0.0004; *B2M, P* = 0.005) and for *ANGPT1* with *B2M* (*P* = 0.013). For *SELL,* a trend for down regulation was observed with *B2M* (*P* = 0.08) and *ACTB* (*P* = 0.11). For *AOC3,* a trend for down regulation was observed only with *B2M* (P = 0.08). For *CD86*, the significant differences in expression levels revealed by microarray analysis were not detected by qRT-PCR with both *ACTB* and *B2M*. This inconsistency between microarray and qRT-PCR data could derive from the several protein coding transcripts of *CD86* gene (http://www.ensembl.org/Homo_sapiens/Gene/Summary?db=core;g=ENSG00000114013;r=3:122055366-122121139), two of which are recognized by the microarray probe in the 3’ UTR and six (four additional transcripts) are potentially amplified by the q-PCR primers, bridging the last exons. With the exception of *CD86*, the expression regulation (up or down) in MS- vs control jugular walls indicated by microarray analysis was supported by qRT-PCR analysis (Additional file 3: Tables S3 and Additional file 4: Table S4).

### Analysis of protein levels in jugular and peripheral plasma

In order to investigate whether differences in the transcriptome profiles between MS- and control jugular walls would correlate with differences in protein expression levels, we selected genes whose protein products could be measured in plasma, and particularly by using a multiplex detection approach. Ten candidate proteins were eligible (Additional file 5: Table S5). In addition, CD86, in the list of the top ten most significantly up regulated coding genes and showing the highest fold-change, was measured as soluble antigen in plasma by a single ELISA.

The selected proteins mainly participate in adhesion (NCAM1, VAP1, SELL), which was among the most significantly enriched process revealed by jugular wall transcriptome analysis, immune/inflammatory responses (CD86, TNF, TNFRSF6B, CCL3, CCL13, CCL18), angiogenesis (ANGPT1) and cytoskeleton/organelle organization (MAPT).

Protein levels were evaluated in jugular and peripheral plasma from 17 patients (MS 1st population) and in peripheral plasma from 34 healthy subjects (Table 2).

**Table 2:** Protein plasma levels in jugular vein (1st MS population) and in peripheral vein (1st MS population and healthy subjects)

PROTEINS	MS/CCSVI JUGULAR PLASMA (*n* = 17)	P*	MS/CCSVI PERIPHERAL PLASMA (*n* = 17)	*P* ^#^	HEALTHY PERIPHERAL PLASMA (*n* = 34)	mRNA PROFILING
ANGPT1	2.6 ± 0.90	0.016	3.6 ± 1.7	0.02	6.2 ± 2.8	↓
CCL13	87.3 ± 32.8	0.22	79.6 ± 23.2	0.33	89.7 ± 39.6	↓
CCL18	27.2 ± 9.3	0.048	30.4 ± 11.1	0.30	34.7 ± 15.5	↓
CD86	183.9 ± 50.7	0.004	221.3 ± 66.4	0.7	214.2 ± 62.4^§^	↑
NCAM1	138.2 ± 57	0.005	149.9 ± 58.3	0.08	123.5 ± 44.2	↑
SELL	451.4 ± 97.5	0.002	522.7 ± 117.2	0.16	584.4 ± 158.8	↑
VAP1	223.8 ± 45.6	0.06	241.7 ± 43.5	0.09	272.0 ± 65.8	↓

Proteins: *ANGPT1* angiopoietin 1, *CCL13* chemokine ligand 13, *CCL18* chemokine ligand 18, *CD86* cluster of differentiation 86, *NCAM1* neural cell adhesion molecule 1, *SELL* selectin L, *VAP1(AOC3)* vascular adhesion protein 1(amine oxidase copper containing 3). Protein concentrations are reported in ng/ml, except for CD86 (U/ml). All values are expressed as mean ± standard deviation. Arrows indicate up (↑) or down (**↓)** mRNA regulation in MS vs non-MS jugular wall. *P values from paired t-test (MS jugular plasma vs MS peripheral plasma). ^#^*P* value from t-test on peripheral plasma (MS patients vs healthy subjects). § evaluated in 28 plasma controls

The comparison of peripheral plasma levels between MS patients and healthy subjects showed significant differences for ANGPT1 (3.6 ± 1.7 vs 6.2 ± 2.8 ng/ml, *P* = 0.02). For NCAM1 and VAP1, the *P* values (0.08 and 0.09 respectively) suggested a trend for differences between patients and healthy subjects.

The lower levels of ANGPT1 and VAP1, and the trend for higher levels of NCAM1in patients, might mimic the RNA expression regulation in the MS jugular wall estimated by transcriptomic analysis (Table 2).

**Fig. 2:**
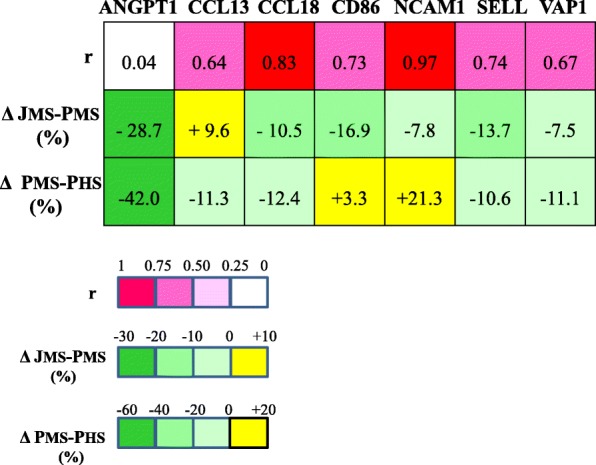
Correlations and variations in protein plasma levels in the 1th MS population. r, Pearson coefficient of the correlation between jugular and peripheral plasma levels in MS patients. Δ JMS-PMS %, percentage difference between jugular and peripheral (100%) plasma levels in MS patients. Δ PMS-PHS %, percentage difference between MS and healthy (100%) peripheral plasma levels

In MS patients, correlation between jugular vein and peripheral plasma concentrations ranged from very high (*r* = 0.97, NCAM1) to virtually absent (*r* = 0.04, ANGPT1) (Fig. 2). Concentrations of ANGPT1, CD86, NCAM1 and SELL were significantly lower (paired t-test) in jugular than in peripheral plasma, with ANGPT1 showing the highest percentage difference (Δ = − 28.7%, Fig. 2).

Four proteins (CCL3, MAPT, TNF and TNFRSF6B) resulted undetectable in the majority of plasma samples in the multiplex assay condition (data not shown).

### Analysis of protein levels in peripheral plasma - 2nd MS population

Peripheral plasma levels of ANGPT1, CCL13, CCL18, NCAM1, SELL and VAP1 were further analysed in an independent MS population (2nd study population). Levels were investigated in 60 patients, grouped by PP-MS and SP-MS clinical phenotypes (Additional file 6: Table S6), and over 4 time points in 56 of them (Table 3). Peripheral plasma protein levels of the 2nd MS population were compared with those of the 1st MS population and of healthy subjects (Table 4).

**Table 3:** Protein plasma levels over four time points in the 2nd MS population

	Time points					
	T0	T1	T2	T3	*P*-value	r
ANGPT1	6.3 ± 4	5.6 ± 3	6.3 ± 3.5	6.6 ± 4.1	0.186	0.675
CCL13	113.9 ± 53.6	108.2 ± 44.7	111.8 ± 44.4	116.5 ± 50.5	0.266	0.832
CCL18	44.6 ± 20.8	43.6 ± 20.5	44.9 ± 20.5	43.9 ± 21.9	0.568	0.945
NCAM1	137.5 ± 56.3	135.3 ± 54.2	133.4 ± 53.8	135.2 ± 58	0.137	0.980
SELL	553.2 ± 115.4	553 ± 113	527.6 ± 105.2	512.3 ± 103.9	< 0.0001	0.897
VAP1	315.6 ± 84.8	306 ± 81.8	310.5 ± 82.5	310.1 ± 81.4	0.383	0.905

Protein levels were evaluated in 56/60 patients. Protein abbreviations are reported as in Table 2

The *P* value of ANOVA for repeated measures across time is reported

r = Pearson coefficient of correlations across 4 time points

**Table 4:** Comparison of protein plasma levels in MS patients (1st and 2nd populations) and healthy subjects

	2^nd^ Population vs 1^st^ Population		2^nd^ Population vs Healthy subjects	
	t-test	ANCOVA	t-test	ANCOVA
ANGPT1	0.021	0.033	0.261	0.330
CCL13	0.021	0.167	0.013	0.690
CCL18	0.004	0.082	0.002	0.302
NCAM1	0.231	0.332	0.204	0.050
SELL	0.241	0.079	0.914	0.161
VAP1	0.001	0.025	0.007	0.389

Protein abbreviations are reported as in Table 2. Protein levels were evaluated in 42 healthy subjects

The *P* values of t-test and ANCOVA (using age as covariate) are reported

No differences in plasma protein levels between clinical subgroups, PP-MS and SP-MS were detected either at time 0 (Additional file 6: Table S6) or overtime (data not reported). As significant age differences were present among clinical groups (PP-MS vs SP-MS, *P* < 0.001), ANCOVA adjusted for age was used to evaluate plasma levels, which did not reveal differences (Additional file 6: Table S6).

The comparison between the 2nd MS population and healthy subjects (Table 4) showed, after t-test, significant differences for CCL13, CCL18 and VAP1. However, after correction for age, only NCAM1 showed higher levels in MS patients than healthy subjects (137.3 ± 54.5 ng/mL vs. 124 ± 44 ng/mL; *P* = 0.050).

The comparison between the 1st and 2nd MS populations (Table 4) showed significant differences (t-test) for AGPT1, CCL13, CCL18 and VAP1. After correction for age significant differences were observed for ANGPT1 and VAP1, and as a trend for CCL18 and SELL.

The analysis overtime of plasma protein levels in the 2nd population, aimed at evaluating the stability of protein levels in plasma, detected a significant difference over time only for SELL (*P* < 0.0001). In particular, pairwise analysis revealed differences between several time points (T0-T1, q = 0.023; T0-T2, q = 0.011); T0-T3, q < 0.0001; T1-T3, q = 0.048). High correlation among time points for each protein was observed, ranging from *r* = 0.67 (ANGPT1) to *r* = 0.98, the noteworthy value for NCAM1.

**Fig. 3:**
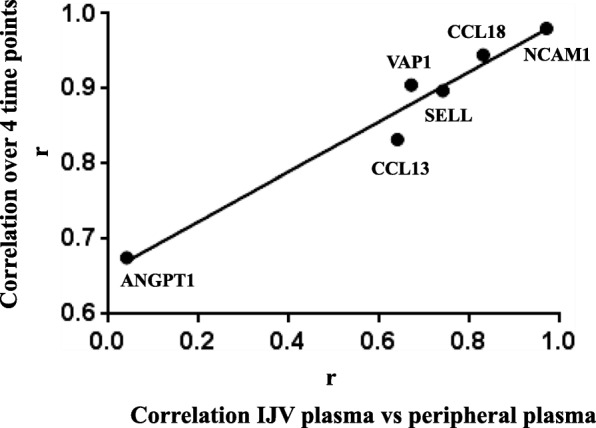
Correlations of protein plasma levels: relation between 1st and 2nd MS population values. X axis: Pearson coefficients (r) of the correlation between jugular and peripheral plasma in 1st MS population. Y axis: Pearson coefficients (r) of the correlation over 4 time points in the peripheral plasma of the 2nd MS population

Repeated protein assays in the 1st MS population (jugular and peripheral plasma) and in the 2nd MS population (four time points in peripheral plasma) offered the opportunity to compare, in independent experiments, concentration variation between vascular bed compartments and overtime. The relation between correlation coefficients is shown in Fig. 3. Interestingly, the r values between jugular and peripheral plasma protein concentrations in the 1st MS population, which ranged from virtually absent (*r* = 0.04, ANGPT1) to very high (*r* = 0.97, NCAM1, Fig. 2), and those observed overtime in the 2nd MS population (Table 3) were highly correlated (R^2^ = 0.96, *P* < 0.001, r Pearson =0.981, Fig. 3).

## Discussion

Early and recent observations (Karmon et al. 2012) suggest interactions between vascular abnormalities and neurodegenerative component in the manifestations of MS, which supports the investigation of both circulating and wall associated factors. We aimed at contributing to these issues, in particular to the involvement of venous compartment, both by transcriptome and plasma protein investigation in MS patients. Surgical reconstruction of malformed IJV in patients was instrumental for the analysis of transcriptome of the jugular vein wall, which in our knowledge has never been performed.

In transcriptomic profiling, confirmed by qRT-PCR, *L1CAM* emerged as the most significantly downregulated gene, among several coding for proteins participating to adhesion processes. This neural cell adhesion molecule has been shown to function in a variety of dynamic neurological processes and to support adhesion by multiple vascular and platelet integrins (Felding-Habermann et al. 1997) with implication in vascular processes.

Noticeably, transcriptomic data indicated dysregulation of the “pattern specification” and “nucleosome organization” processes, that could be related to the altered features of jugular flow observed in MS patients with associated chronic cerebrospinal venous insufficiency. Several members of the HOX transcription factors family, overexpressed in patients’ jugular wall transcriptome and belonging to the “pattern specification” process, are known to regulate embryogenesis, development and also processes in adult tissues, among which vasculature pathways (Gorski and Walsh 2003). In particular, *HOXA5*, and *HOXB5* have been found to be blood flow-sensitive in endothelial cells (Passerini et al. 2004). Disturbed flow conditions have been found to affect also expression of histone genes (“nucleosome organization”) in cultured endothelial cells from human carotid artery (Aoki et al. 2016). Our findings link altered transcriptional profiles of the jugular wall in patients to a number of important experimental observations obtained at the gene expression level in cellular and animal models unrelated to MS. Further, the down- regulation of several genes, related to muscle contraction, muscular/cytoskeleton system and members of the large collagen family, might be related to altered features and properties of the internal jugular vein, and particularly anatomy, histology and flow abnormalities (Zamboni et al. 2009; Coen et al. 2013b). However, only the availability of jugular wall expression profiling from MS patients not meeting the criteria for CCSVI, an unattainable goal, would permit to specifically attribute expression changes to MS or to vascular changes in MS- related CCSVI.

The strong up-regulation in patients’ jugular wall transcriptome of CD86, a costimulatory protein involved in several mechanisms of immune response (Jeannin et al. 1999), could be related to immune activation at the level of jugular wall, potentially including immune cells adhering to the vessel surface, coherently with the well-known autoimmune features of MS. Remarkably, overexpression of CD86 transcripts in PBMC at all the stages of MS compared with healthy controls was recently reported (Srinivasan et al. 2017).

Altogether the transcriptome analysis in jugular wall and the transcriptome analysis in PBMC from MS patients (Comabella et al. 2016; Srinivasan et al. 2017; Iglesias et al. 2004) suggest dysregulation of histone, cytoskeleton and CD86 genes as a general signature of altered gene expression in different cells and tissues of MS patients.

Investigation of changes observed at RNA level was combined with that in plasma at the protein level, through analysis of molecules acting in immune-inflammatory, in cell adhesion/neuronal cell adhesion and angiogenesis processes, all known to play a role in MS pathogenesis. (Salmi and Jalkanen 2017).

In addition to the 1st population of MS/CCSVI patients a 2nd population with progressive MS phenotypes was analysed for deeper investigation at the plasma protein level and to increase robustness of our study. The repeated measurements help to define particularly conserved plasma patterns, well exemplified by the statistical analysis of CCL18 and VAP-1 values (Table 3).

VAP1, an amine oxidase with also adhesive activities (Salmi and Jalkanen 2017), involved in a rat model of MS in CNS inflammatory lesion development (Elo et al. 2018), was previously found to be significantly lower in RR and in SP patients with absence of MRI active lesions than in controls (Airas et al. 2006). The Finnish cohort mirrors the 1st MS population of our study, in which the majority of patients were free from gadolinium enhancing lesions at preoperative MRI (Table 1), and thus with absence of ongoing inflammatory activity within the brain. Further, the analysis conducted in the 2nd MS population clearly indicated both age and disease phenotypes as important determinants of VAP1 plasma levels.

To further characterize features of the IJV compartment in patients, the concentration of several proteins was analysed in paired jugular and peripheral vein plasma samples. For most molecules, the good to excellent jugular-peripheral correlations, which were highly related to those observed overtime in the 2nd MS population, support the quality of our analysis, conducted by a multiplex assay that prevents most of bias in experimental condition among protein antigens. This approach also permitted to compare variation between vascular compartments with variation over time of specific protein levels. Protein biosynthesis, bio-distribution and stability could participate in producing the different extent of correlation observed for each protein.

Noticeably, significantly lower levels in jugular were assessed for most proteins, with CCL13 being the only exception. The paucity of studies in literature, comparing jugular and peripheral plasma profiles in MS patients, and the unavailability of jugular plasma from healthy individuals limit the interpretation of the observed differences.

Although NCAM1 is thought to be involved in several processes, like neuronal development, organization of synapses and myelination/remyelination process, that take place in MS (Massaro 2002), data concerning plasma levels of NCAM1 in MS patients are not available in literature. In our study, both MS populations showed as a trend higher levels than healthy subjects. Taking into account the tight correlations (*r* = 0.97) between jugular and peripheral plasma levels of NCAM1 and among repeated evaluations over time (*r* = 0.98) performed in the 2nd MS population, which indicate the presence of particularly conserved individual plasma patterns in two MS disease cohorts, our findings suggest that NCAM1 plasma levels could be related to MS disease, independently from the CCSVI status.

Finding significant SELL level variation overtime, in presence of high correlation, could highlight persisting changes dependent on the rehabilitative treatment in patients, as inferred by decreasing SELL concentrations from T0 to T3 time points.

ANGPT1, investigated for the first time in plasma of MS patients, showed remarkably lower levels in the jugular vein than in peripheral plasma. ANGPT1 levels in the 2nd MS population was similar to those in healthy subjects and higher than in the 1st MS population, even after correction for age, which suggests a CCSVI-related association. Further, the absence of correlation between values in jugular/peripheral compartments (*r* = 0.04), compared with the good correlation estimated in repeated overtime measurements (*r* = 0.67), would support the presence of specific mechanisms regulating ANGPT1 levels and, as jugular vein drains blood from brain, cerebral expression/uptake might be candidate. The lower levels of ANGPT1 in plasma from the first MS population might mimic the lower RNA expression in the MS jugular wall estimated by transcriptomic analysis.

Intriguingly, ANGPT1 is thought to play an essential role in microvascular endothelial and blood-brain barrier integrity. Indeed, ANGPT1, produced by endothelial cells, through the Tie-2 receptor is implicated on blood vessel stability and integrity by inhibiting blood vessel leakage, and reducing the infiltration of inflammatory cells (Thurston et al. 2000; Lee et al. 2011). This protective role of ANGPT1 has been suggested in experimental allergic encephalomyelitis studies (Wang et al. 2016), and interestingly mutations in the Tie-2 receptor were found to be associated with venous malformations (Nätynki et al. 2015).

The “long way” from jugular wall RNA to plasma protein is a remarkable limitation to study the parallel between mRNA and plasma protein concentrations. As a matter of fact, for CD86 several transcripts have been reported and the plasma assay (Wong et al. 2005) is able to detect only the soluble protein form of this membrane receptor, produced either by shedding or by alternative mRNA splicing. These CD86 mRNA and protein features have prevented detection of relation between mRNA and protein expression. Our study presents other limitations. First, the small number of vascular wall specimens undergoing transcriptomic analysis, tiny specimens of internal jugular wall, which represent by necessity very rare samples. Another limitation concerns the “control” CEA samples, being virtually unavailable jugular samples (wall) from MS patients, not meeting criteria for CCSVI, and from healthy individuals. Nevertheless, the analysis of protein levels in peripheral plasma in two independent MS populations, and in addition through repeated assays, favored investigation of MS- related variations.

## Conclusions

Our study provides for the first time expression profiles of the IJV wall and suggests signatures of altered vascular mRNA profiles in MS disease. Repeated measurements in plasma indicate conserved plasma patterns for immune-inflammatory and adhesion proteins. The combined transcriptome-protein analysis provides intriguing links between IJV wall transcript alteration and plasma protein expression, thus highlighting proteins of interest for MS pathophysiology.

## Additional files

Additional file 1 **Table S1.** Demographics and clinical characteristics of the 2nd study MS population. (DOCX 16 kb)

Additional file 2 **Table S2.** qRT-PCR Forward and Reverse Primers. (DOCX 15 kb)

Additional file 3 **Table S3**. List of 924 transcripts/genes differentially expressed (fold change > or = 2, Benjamini-Hockberg corrected *P* value < 0.05) in internal jugular vein wall of MS patients (MS-IJW) compared to non-MS controls (C-IJW). (XLSX 207 kb)

Additional file 4 **Table S4**. qRT-PCR expression levels of selected genes in MS (*n* = 7) vs control (*n* = 4) jugular vein walls. (DOCX 21 kb)

Additional file 5 **Table S5**. List of genes, differentially expressed in MS jugular vein walls (MS-IJW) compared to control vein walls (C-IJW), selected for protein level analysis in plasma. (DOCX 22 kb)

Additional file 6 **Table S6**. Plasma protein levels in the 2nd MS population according to clinical phenotypes. (DOCX 18 kb)

## Abbreviations

ACTB: Actin beta
ANGPT1: Angiopoietin 1
AOC3 (or VAP-1): Amine oxidase copper containing 3 (or vascular adhesion protein 1)
B2M: Beta-2-microglobulin
CCL13: C-C motif chemokine ligand 13
CCL18: C-C motif chemokine ligand 18
CCL3: C-C motif chemokine ligand 3
CCSVI: chronic cerebrospinal venous insufficiency
CD86: Cluster of differentiation 86
EDSS: Expanded disability status scale
GO: Gene ontology
HOX: Homeobox
IJV: Internal jugular vein
L1CAM: Neural cell adhesion molecule L1
MAPT: Microtubule associated protein tau
MRI: Magnetic resonance imaging
MS: Multiple sclerosis
NCAM1: Neural cell adhesion molecule1
PBMCs: Peripheral blood mononuclear cells
PP-MS: Primary progressive multiple sclerosis
RR-MS: Relapsing remitting multiple sclerosis
SELL: Selectin L
SP-MS: Secondary progressive multiple sclerosis
TNF: Tumor necrosis factor
TNFRSF6B: TNF receptor superfamily member 6b
