# Supplementary material for: Changes in expression profiles of internal jugular vein wall and plasma protein levels in multiple sclerosis
Source: Mol Med. 2018 Aug 9;24:42. doi: 10.1186/s10020-018-0043-4 (PMC6085618; doi:10.1186/s10020-018-0043-4)
Supplement: Supplementary file 1 — Table S1. Demographics and clinical characteristics of the 2nd study MS population. (DOCX 16 kb) [file 10020_2018_43_MOESM1_ESM.docx]

**Table S1.** Demographics and clinical characteristics of the 2^nd^ study MS population.

|  | All cohort  n= 60 | PP-MS  n= 28 | SP-MS  n= 32 |
| --- | --- | --- | --- |
|  |  |  |  |
| Age, mean ± SD | 55.5 ± 10.5 | 58.5 ± 11.1 | 52.8 ± 9.3 |
| Gender, M/F | 21/39 | 6/22 | 15/17 |
| Disease duration, mean ± SD | 15.1 ± 10.5 | 12.6 ± 11.7 | 17.35 ± 8.7 |
| EDSS, mean ± SD | 6 ± 0.5 | 6 ± 0.5 | 6.5 ± 0.5 |

RR, relapsing remitting; PP, primary progressive; SP, secondary progressive; EDSS, expanded disability status scale. Age and disease duration are reported in years.
