# Supplementary material for: Changes in expression profiles of internal jugular vein wall and plasma protein levels in multiple sclerosis
Source: Mol Med. 2018 Aug 9;24:42. doi: 10.1186/s10020-018-0043-4 (PMC6085618; doi:10.1186/s10020-018-0043-4)
Supplement: Supplementary file 2 — Table S2. qRT-PCR Forward and Reverse Primers. (DOCX 15 kb) [file 10020_2018_43_MOESM2_ESM.docx]

**Table S2.** qRT-PCR Forward and Reverse Primers.

| **GENE** | **SEQUENCE** |
| --- | --- |
| ***ANGPT1*** | 5’- GAGGATGGTGGTTTGATGCT-3’  5’- CAATATTGTTTGCTTCTGAAGTTTTC-3’ |
| ***AOC3*** | 5’- TACCAGCTGGCTGTGACC-3’  5’- TGGGATATGCAGAAAACCAG-3’ |
| ***CD86*** | 5’- GGAAGAGAGTGAACAGACCAAGA-3’  5’- AATGTCTTTTTGCCTTCTGGA- 3’ |
| ***L1CAM*** | 5’- AGGACACCCAGGTGGACTC-3’  5’- TCCTCGTTGAACTGAACATCC-3’ |
| ***SELL*** | 5’- CTCTGGGTTGGCATTTATCA-3’  5’- CTTCCCAGATGCACTGAAGG-3’ |
| ***ACTB**** | 5’- CATCGAGCACGGCATCGTCA-3’  5’- TAGCACAGCCTGGATAGCAAC-3’ |
| ***B2M**** | 5’- TTTCATCCATCCGACATTGA-3’  5’- CCTCCATGATGCTGCTTACA-3’ |

* endogenous controls.
