# Supplementary material for: Changes in expression profiles of internal jugular vein wall and plasma protein levels in multiple sclerosis
Source: Mol Med. 2018 Aug 9;24:42. doi: 10.1186/s10020-018-0043-4 (PMC6085618; doi:10.1186/s10020-018-0043-4)
Supplement: Supplementary file 4 — Table S4. qRT-PCR expression levels of selected genes in MS (n = 7) vs control (n = 4) jugular vein walls. (DOCX 21 kb) [file 10020_2018_43_MOESM4_ESM.docx]

**Table S4**. qRT-PCR expression levels of selected genes in MS (n=7) vs control (n=4) jugular vein walls.

| Gene symbol | Description | Fold-change  mean ±SEM  *B2M* | P | Fold-change  mean ±SEM  *ACTB* | P | Regulation  in microarray |
| --- | --- | --- | --- | --- | --- | --- |
| *ANGPT1*^$^ | Angiopoietin1 | 0.60 ± 0.10 | 0.013 | 0.64 ± 0.13 | 0.13 | down |
| *AOC3 ^&^ (VAP-1)* | Amine oxidase copper containing 3 | 0.45 ± 0.12 | 0.08 | 0.82 ± 0.14 | 0.31 | down |
| *CD86* ^#^ | Cluster of differentiation 86 | 1.21 ± 0.17 | 0.55 | 1.67 ± 0.35 | 0.22 | up |
| *L1CAM*^&^ | L1 cell adhesion molecule | 0.46 ± 0.09 | 0.0052 | 0.33 ± 0.06 | 0.0004 | down |
| *SELL*^&^ | Selectin L | 2.08 ± 0.57 | 0.08 | 1.88 ± 0.51 | 0.11 | up |

Expression values obtained by qRT-PCR are reported as mean fold change ± standard error of the mean (SEM). *B2M* (beta-2-microglobulin) and *ACTB* (actin beta ) were used as endogenous control genes. The P values from t-test are reported. *AOC3* is also known as *VAP-1*, vascular adhesion protein 1. Main processes from Gene Ontology database: ^#^ immune/inflammatory response, ^$^angiogenesis,  ^&^adhesion.
