# Supplementary material for: Changes in expression profiles of internal jugular vein wall and plasma protein levels in multiple sclerosis
Source: Mol Med. 2018 Aug 9;24:42. doi: 10.1186/s10020-018-0043-4 (PMC6085618; doi:10.1186/s10020-018-0043-4)
Supplement: Supplementary file 5 — Table S5. List of genes, differentially expressed in MS jugular vein walls (MS-IJW) compared to control vein walls (C-IJW), selected for protein level analysis in plasma. (DOCX 22 kb) [file 10020_2018_43_MOESM5_ESM.docx]

**Table S5**. List of genes, differentially expressed in MS jugular vein walls (MS-IJW) compared to control vein walls (C-IJW), selected for protein level analysis in plasma.

| Gene symbol | Description | Regulation | Expression  MS-IJW  mean ± SD | Expression  C-IJW  mean ± SD | Fold  change | P value* |
| --- | --- | --- | --- | --- | --- | --- |
|  |  |  |  |  |  |  |
| *CD86* ^#^ | Cluster of differentiation 86 | up | 14.38 ± 0.89 | 11.30 ± 0.38 | 8.47 | 0.002 |
| *ANGPT1*^$^ | Angiopoietin1 | down | 9.20 ± 0.14 | 10.48 ± 0.29 | 2.02 | 0.002 |
| *CCL18* ^#^ | C-C motif chemokine ligand 18 | down | 8.50 ± 0.41 | 10.99 ± 0.88 | 5.60 | 0.005 |
| *TNF* ^#^ | Tumor necrosis factor | up | 9.21 ± 1.24 | 6.34 ± 0.76 | 7.31 | 0.009 |
| *NCAM1*^&^ | Neural cell adhesion molecule 1 | up | 4.00 ± 0.35 | 2.57 ± 0.70 | 2.69 | 0.016 |
| *TNFRSF6B* ^#^ | TNF receptor superfamily member 6b | down | 6.33 ± 0.62 | 7.48 ± 0.33 | 2.22 | 0.017 |
| *AOC3 ^&^ (VAP-1)* | Amine oxidase copper containing 3 | down | 11.54 ± 1.29 | 13.85 ± 0.78 | 4.94 | 0.020 |
| *CCL13* ^#^ | C-C motif chemokine ligand 13 | down | 6.78 ± 1.28 | 9.28 ± 1.12 | 5.66 | 0.024 |
| *CCL3* ^#^ | C-C motif chemokine ligand 3 | up | 14.04 ± 1.54 | 9.83 ± 2.64 | 18.43 | 0.032 |
| *SELL*^&^ | Selectin L | up | 9.34 ± 0.73 | 7.86 ± 0.98 | 2.78 | 0.045 |
| *MAPT ^£^* | Microtubule associated protein tau | down | 7.22 ± 0.35 | 8.35 ± 0.86 | 2.19 | 0.048 |

Genes are ordered according to differential expression P values. * by moderate t-test, followed by the application of Benjamini- Hoechberg multiple testing correction. The mean ± SD of the log-transformed (log2) expression values is reported. The fold change is presented as an absolute value. Main processes from Gene Ontology database: ^#^ immune/inflammatory response, ^$^angiogenesis,  ^&^adhesion, ^£^ cytoskeleton organization. *AOC3* is also known as *VAP-1*, vascular adhesion protein 1.
