# Supplementary material for: Changes in expression profiles of internal jugular vein wall and plasma protein levels in multiple sclerosis
Source: Mol Med. 2018 Aug 9;24:42. doi: 10.1186/s10020-018-0043-4 (PMC6085618; doi:10.1186/s10020-018-0043-4)
Supplement: Supplementary file 6 — Table S6. Plasma protein levels in the 2nd MS population according to clinical phenotypes. (DOCX 18 kb) [file 10020_2018_43_MOESM6_ESM.docx]

**Table S6**. Plasma protein levels in the 2^nd^ MS population according to clinical phenotypes.

|  | **MS PP-MS vs SP-MS** | | | | |
| --- | --- | --- | --- | --- | --- |
|  |  | **PP-MS** | **SP-MS** | t-test  P-value | ANCOVA  P-value |
|  | n=60 | n=28 | n=32 |  |  |
| **ANGPT1** | 6.4 ± 4 | 6.5 ± 3.6 | 6.4 ± 4.4 | 0.941 | 0.974 |
| **CCL13** | 116.3 ± 54.7 | 126.2 ± 64.6 | 107.6 ± 43.6 | 0.191 | 0.417 |
| **CCL18** | 44.6 ± 20.2 | 48.6 ± 24.2 | 41.1 ± 15.6 | 0.152 | 0.495 |
| **NCAM1** | 137.3 ± 54.5 | 145.3 ± 64.6 | 130.3 ± 43.9 | 0.292 | 0.189 |
| **SELL** | 558.6 ± 118.2 | 568.9 ± 118.6 | 549.6 ± 119 | 0.531 | 0.340 |
| **VAP1** | 313.8 ± 83.5 | 312.4 ± 77 | 315.1 ± 90 | 0.902 | 0.417 |

PP-MS: primary progressive multiple sclerosis; SP-MS: secondary progressive multiple sclerosis. The P values of t-test and ANCOVA (using age as covariate) are reported.

Proteins: ANGPT1, angiopoietin 1; CCL13, chemokine ligand 13; CCL18, chemokine ligand 18; NCAM1, neural cell adhesion molecule 1; SELL, selectin L; VAP1(AOC3), vascular adhesion protein 1(amine oxidase copper containing 3).
